# Peculiar evolution of streptococcal skin infections

**DOI:** 10.1186/1471-2334-13-S1-P66

**Published:** 2013-12-16

**Authors:** Sonia Drăghici, Andrei Csep

**Affiliations:** 1 Faculty of Medicine and Pharmacy, University of Oradea, Romania

## Background

Acute *Streptococcus pyogenes* infections may present under various forms, such as pharyngitis, scarlet fever, pneumonia, meningitis, cellulitis or impetigo. Toxigenic infections can result in necrotizing fasciitis, myositis and streptococcal toxic shock syndrome. Following acute infections caused by *S pyogenes*, patients are also at risk for developing immune-mediated post-streptococcal sequelae, as is the case with acute rheumatic fever and acute glomerulonephritis.

## Methods

We performed a hospital based study from January 2012 to December 2012 in the Clinic of Infectious Diseases Oradea.

## Results

A total number of 192 patients were studied during a one-year period: 25 cases of impetigo and erysipelas, in parallel with 84 cases of tonsillitis, 36 cases of scarlet fever, 10 cases of acute rheumatic fever, 8 cases of chronic rheumatic heart disease, 6 cases of acute post streptococcal glomerulonephritis, 2 cases of streptococcal meningitis, and 31 cases of healthy carriers of *S pyogenes*.

Impetigo dominated in children (7 cases – 70%) vs. adults (3 cases – 30%); erysipelas dominated in adults, especially over 40 years (13 cases – 86%) vs. children (2 cases – 14%). From the 25 patients admitted in the hospital with the diagnosis of erysipelas, 11 patients (60%) declared one or more (5) recurrences in the same part of the body. In rheumatic fever, approximately 80% of the patients will have an elevated ASO titer (>200 Todd units) at 2 months after onset.

## Conclusion

The type and severity of streptococcal infections depends on the strain of *S pyogenes* and on the comorbid conditions or diseases, like heart, pulmonary, renal, metabolic chronic diseases, obesity or lymphatic stasis.

The septic, toxic and the immune complications of streptococcal infections pose important problems for the public health services.

